# Investigation on Gold–Ligand Interaction for Complexes from Gold Leaching: A DFT Study

**DOI:** 10.3390/molecules28031508

**Published:** 2023-02-03

**Authors:** Na Zhang, Jue Kou, Chunbao Sun

**Affiliations:** School of Civil and Resource Engineering, University of Science and Technology Beijing, Beijing 100083, China

**Keywords:** gold leaching, gold complex, DFT, CDA, EDA

## Abstract

Gold leaching is an important process to extract gold from ore. Conventional alkaline cyanide process and alternative nontoxic lixiviants including thiosulfate, thiourea, thiocyanate, and halogen have been widely investigated. However, density functional theory (DFT) study on the gold complexes Au(CN)_2_^−^, Au(S_2_O_3_)_2_^3−^, Au[SC(NH_2_)_2_]_2_^+^, Au(SCN)_2_^−^, and AuCl_2_^−^ required for discovering and designing new highly efficient and environmentally friendly gold leaching reagents is lacking, which is expected to support constructive information for the discovery and designation of new high-efficiency and environmentally friendly gold leaching reagents. In this study, the structure information, electron-transferring properties, orbital interaction, and chemical bond composition for complexes Au(CN)_2_^−^, Au(S_2_O_3_)_2_^3−^, Au[SC(NH_2_)_2_]_2_^+^, Au(SCN)_2_^−^, and AuCl_2_^−^ depending on charge decomposition analysis (CDA), natural bond orbital (NBO), natural resonance theory (NRT), electron localization function (ELF), and energy decomposition analysis (EDA) were performed based on DFT calculation. The results indicate that there is not only σ-donation from ligand to Au^+^, but also electron backdonation from Au^+^ to ligands, which strengthens the coordinate bond between them. Compared with Cl^−^, ligands CN^−^, S_2_O_3_^2−^, SC(NH_2_)_2_, and SCN^−^ have very large covalent contribution to the coordinate bond with Au^+^, which explains the special stability of Au-CN and Au-S bonds. The degree of covalency and bond energy in Au–ligand bonding decreases from Au(CN)_2_^−^, Au(S_2_O_3_)_2_^3−^, Au[SC(NH_2_)_2_]_2_^+^, Au(SCN)_2_^−^, to AuCl_2_^−^, which interprets the stability of the five complexes: Au(CN)_2_^−^ > Au(S_2_O_3_)_2_^3−^ > Au[SC(NH_2_)_2_]_2_^+^ > Au(SCN)_2_^−^ > AuCl_2_^−^.

## 1. Introduction

As one of the most important noble metals, gold is not only used as an ornament, a concentrated form of wealth, and money, it also has critical technological applications in the electronic, computer, and space fields [1]. To meet the use of gold in society, gold extraction has shown its importance. Au possesses a nontoxic nature, stability against metal leaching, and resistance to excessive oxidation by O_2_ [2]. However, gold becomes unstable and dissolves in the positive potential region of electrochemical processes, especially in solutions containing CN^−^, Cl^−^, and sulfur-containing species such as S_2_O_3_^2−^, SC(NH_2_)_2_, and SCN^−^, which make the extraction of gold from ore feasible [3,4,5].

The conventional alkaline cyanide process is the dominant method for gold recovery from ores due to its simplicity and high efficiency. The gold leaching process by cyanide is represented by Equation (1), where complex Au(CN)_2_^−^ is formed. However, the cyanide process has a limitation due to its environmental issues. In the past decades, alternative nontoxic lixiviants for gold have drawn extensive attention, including thiosulfate (S_2_O_3_^2−^), thiourea (SC(NH_2_)_2_), thiocyanate (SCN^−^), and halogen [6,7,8,9].

(1) $$4\mathrm{Au}+8{\mathrm{CN}}^{-}+O_{2}+2H_{2}O=4\mathrm{Au}{\left(\mathrm{CN}\right)}_{2}^{-}+4{\mathrm{OH}}^{-}$$

Thiosulfate is the most promising candidate since it is environmentally friendly, inexpensive, and has good selectivity towards gold (Equation (2)). Normally, thiosulfate is used with an ammonia–copper solution, since copper sulfate can be a catalyst and ammonia can stabilize thiosulfate. Although it has environmental benefit, it shows a high reagent consumption [10,11,12,13,14]. Thiourea is an organosulfur compound that forms a soluble cationic complex with gold in a sulfuric acid solution. Fe^3+^ is added to facilitate the oxidation of gold (Equation (3)). However, the use of thiourea would cause a high reagent consumption and a surface passivation [1,2,4,7,15]. Compared to cyanide, thiocyanate is far less toxic and is safer for the environment. Compared to thiourea and thiosulfate, thiocyanate leaching systems require lower reagent consumption and cost, and have better stability than thiourea. Fe^3+^ is normally used as the oxidant (Equation (4)). However, the reaction occurs in an acidic environment, which would be corrosive to equipment [8,16,17,18]. Chlorine is also used as an alternative lixiviant for gold due to its high dissolution rate (Equation (5)), but chlorine is strongly corrosive, highly volatile, and hazardous [9,19,20,21]. The stability constants and standard reduction potentials for Au(CN)_2_^−^, Au(S_2_O_3_)_2_^3−^, Au[SC(NH_2_)_2_]_2_^+^, Au(SCN)_2_^−^, and AuCl_2_^−^ are listed in Table 1 [1,5,14]. The higher the stability constant and the lower the standard reduction potential, the more stable the complex is, so the stability of the five complexes is: Au(CN)_2_^−^ > Au(S_2_O_3_)_2_^3−^ > Au[SC(NH_2_)_2_]_2_^+^ > Au(SCN)_2_^−^ > AuCl_2_^−^. Generating a stable gold–ligand complex is the prerequisite for gold leaching.

(2) $$4\mathrm{Au}+8S_{2}O_{3}^{2-}+O_{2}+2H_{2}O=4{\left[\mathrm{Au}{\left(S_{2}O_{3}\right)}_{2}\right]}^{3-}+4{\mathrm{OH}}^{-}$$

(3) $$\mathrm{Au}+2\mathrm{SC}{\left({\mathrm{NH}}_{2}\right)}_{2}+{\mathrm{Fe}}^{3+}=\mathrm{Au}{\left[\mathrm{SC}{\left({\mathrm{NH}}_{2}\right)}_{2}\right]}_{2}^{+}+{\mathrm{Fe}}^{2+}$$

(4) $$\mathrm{Au}+2{\mathrm{SCN}}^{-}+{\mathrm{Fe}}^{3+}=\mathrm{Au}{\left(\mathrm{SCN}\right)}_{2}^{-}+{\mathrm{Fe}}^{2+}$$

(5) $$2\mathrm{Au}+2{\mathrm{Cl}}^{-}+{\mathrm{Cl}}_{2}=2{\mathrm{AuCl}}_{2}^{-}$$

In addition to the alternative lixiviants mentioned above, new highly efficient and environmentally friendly reagents have been discovered or designed, such as ionic liquid, dicyanamide, ferrocyanide, etc. [22,23,24]. However, those new gold leaching reagents are in the preliminary research phase. The bonding properties of Au(CN)_2_^−^, Au(S_2_O_3_)_2_^3−^, Au[SC(NH_2_)_2_]_2_^+^, Au(SCN)_2_^−^, and AuCl_2_^−^ in the quantum chemistry perspective would support instructive information for the discovery and design of new highly efficient and environmentally friendly gold leaching reagents. Although there are many studies on gold leaching by thiosulfate (S_2_O_3_^2−^), thiourea (SC(NH_2_)_2_), thiocyanate (SCN^−^), and halogen, there is a lack of systematic wavefunction analysis of the gold complexes Au(CN)_2_^−^, Au(S_2_O_3_)_2_^3−^, Au[SC(NH_2_)_2_]_2_^+^, Au(SCN)_2_^−^, and AuCl_2_^−^.

Therefore, in this study, the structure information, electron transferring properties, orbital interaction, chemical bond composition, and bond energy for complexes Au(CN)_2_^−^, Au(S_2_O_3_)_2_^3−^, Au[SC(NH_2_)_2_]_2_^+^, Au(SCN)_2_^−^, and AuCl_2_^−^ depending on charge decomposition analysis (CDA), natural bond orbital (NBO), natural resonance theory (NRT), electron localization function (ELF), and energy decomposition analysis (EDA) have been performed based on density functional theory (DFT) calculation, according to which the stability of the five complexes is also analyzed. The information is expected to support constructive instruction to discover the potential gold leaching reagent.

## 2. Results and Discussion

### 2.1. Optimized Geometries of Gold Complexes

**Figure 1:**
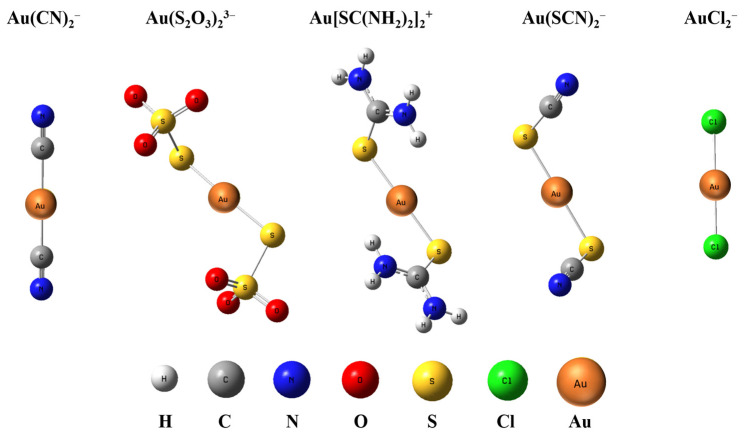
Optimized structure of Au(CN)_2_^−^, Au(S_2_O_3_)_2_^3−^, Au[SC(NH_2_)_2_]_2_^+^, Au(SCN)_2_^−^, and AuCl_2_^−^ at TPSSh/aug-cc-pVTZ level.

The optimized geometries of five gold complexes are shown in Figure 1, whose calculated geometry and structural parameters are listed in Table 2. Au(CN)_2_^−^ and AuCl_2_^−^ have a D_∞h_ symmetry with a linear structure, while Au(S_2_O_3_)_2_^3−^, Au[SC(NH_2_)_2_]_2_^+^, and Au(SCN)_2_^−^ have a C_2_ symmetry where Au^+^ and the ligands are not in the same surface and there is a slight bend for S-Au-S whose angle is around 176~177°. All the bond lengths in the complexes are within the range of the M–X covalent single-bond lengths [25]. The bond lengths from the two levels of theory are consistent with each other within 0.005 Å or better for all the species. The bond lengths and angles of Au-C in Au(CN)_2_^−^, Au-S in Au(SCN)_2_^−^, and Au-Cl in AuCl_2_^−^ at the level of energy-consistent quasi-relativistic pseudopotential basis set TPSSh/aug-cc-pVTZ are consistent with those calculated at the level of scalar relativistic basis set SR-ZORA PBE/TZ2P reported previously [26,27]. The results indicate that the bond length of Au-C in Au(CN)_2_^−^ is the shortest among the four complexes, which is 1.987 Å, and that of Au-X in the other four complexes is similar, which is around 2.3 Å. As bond strength increases, the atoms in the bond are pulled more tightly together. Therefore, the bond energy of Au-C in Au(CN)_2_^−^ is larger than that of Au-S and Au-Cl in other four complexes. The charge population analyses are listed in Table 3. All methods (Hirshfeld, ADCH, CM5, and CHELPG) show that the Au atom carries little charge, while the negative charge is mainly distributed on ligands. The atomic charge on Au is less than 1, indicating that there is electron transfer from ligands to Au atom.

### 2.2. Charge Decomposition Analysis (CDA)

Charge decomposition analysis (CDA) is a partitioning scheme to analyze donor–acceptor interactions. It was developed by Dapprich and Frenking as a quantitative expression of the well-known Dewar–Chatt–Duncanson DCD model, which describes the metal–ligand interactions in terms of donation and backdonation chemical mechanisms [32,33,34]. The CDA method has proven to be very useful for estimating the relative strength of charge donation and backdonation in a series of transition metal complexes [35,36].

Based on CDA, the number of electrons donated from ligands to Au^+^ and the number of electrons backdonated from Au^+^ to ligands are listed in Table 4. It indicates that the coordinate bonds of all five gold complexes are not a pure σ-donation, and there is an electron backdonation from Au^+^ to ligands, although the number of electrons donated from ligands to Au^+^ is larger than that backdonated from Au^+^ to ligands. The backdonation strengthened the bond between Au^+^ and the ligands [37]. The percentage contribution of the backdonation relative to the donation has the tendency to decrease from Au(CN)_2_^−^, Au(S_2_O_3_)_2_^3−^, Au[SC(NH_2_)_2_]_2_^+^, Au(SCN)_2_^−^, to AuCl_2_^−^, which corresponds to the stability of the five complexes. Especially in Au(CN)_2_^−^, its donation and backdonation are both the largest when compared with the other complexes, which would explain its high stability among those complexes.

**Figure 2:**
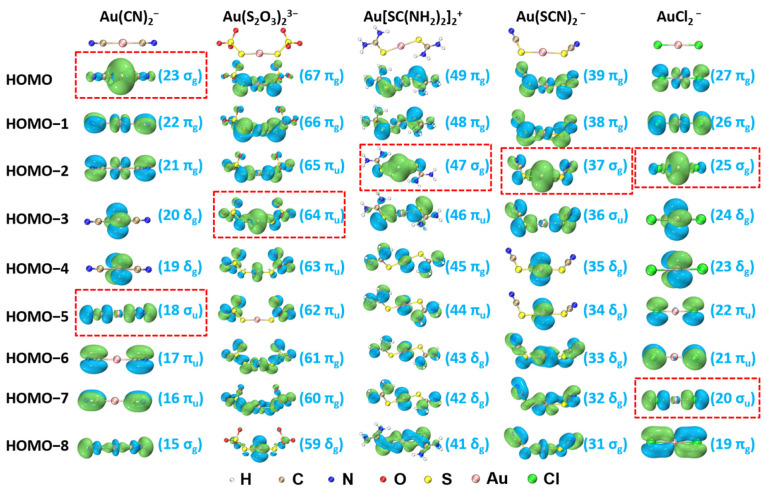
Molecular orbitals (MOs) and corresponding orbital number and type for Au(CN)_2_^−^, Au(S_2_O_3_)_2_^3−^, Au[SC(NH_2_)_2_]_2_^+^, Au(SCN)_2_^−^, and AuCl_2_^−^ (isosurface = 0.03 a.u.). The bonding orbitals are highlighted by red-dash rectangular frames.

The isocontour surfaces of molecular orbitals (MOs) from HOMO to HOMO-8 for Au(CN)_2_^−^, Au(S_2_O_3_)_2_^3−^, Au[SC(NH_2_)_2_]_2_^+^, Au(SCN)_2_^−^, and AuCl_2_^−^ derived from TPSSh/aug-cc-pVTZ calculation are presented in Figure 2. Based on CDA, the main MOs contributing to σ-donations are highlighted by the red-dash rectangular frames shown in Figure 2.

**Figure 3:**
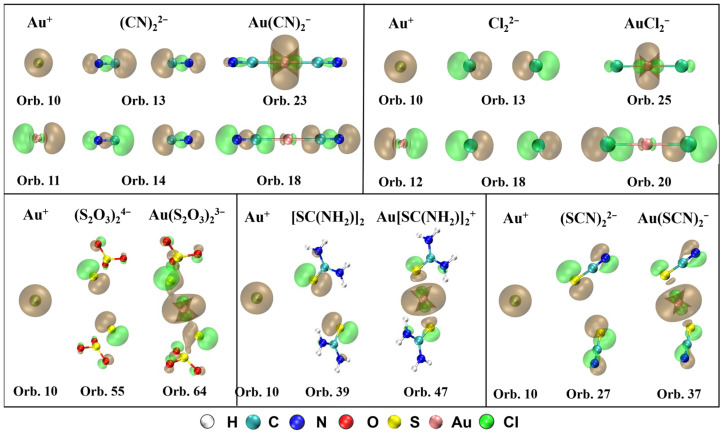
MOs that contribute to σ-donation and their orbital composition from Au^+^ and ligands for Au(CN)_2_^−^, Au(S_2_O_3_)_2_^3−^, Au[SC(NH_2_)_2_]_2_^+^, Au(SCN)_2_**^−^**, and AuCl_2_**^−^** (isosurface = 0.04 a.u.).

The MOs that contribute to σ-donation and their orbital composition from Au^+^ and ligands are shown in Figure 3. It can be observed that the spherical s orbital in Au^+^ participates in the formation of the σ bond for all five complexes. For Au(CN)_2_^−^, there are two orbitals mainly contributing to the σ-bonding between Au^+^ and (CN)_2_^2−^, orbital 23 (HOMO, σ_g_) and orbital 18 (HOMO-5, σ_u_). Orbital 23 is composed of orbital 10 (6s) from Au^+^ and orbital 13 from (CN)_2_^2−^, and orbital 18 consisting of orbital 11 (6p_x_) from Au^+^ and orbital 14 from (CN)_2_^2−^. For Au(S_2_O_3_)_2_^3−^, the number of electrons donated from ligands to Au^+^ is the most in orbital 64 (HOMO-3, π_u_), which is assembled by orbital 10 from Au^+^ and orbital 55 from (S_2_O_3_)_2_^4−^. For Au[SC(NH_2_)_2_]_2_^+^, in orbital 47 (HOMO-2, σ_g_), [SC(NH_2_)_2_]_2_ transfers the most electrons to Au^+^ through the σ-bonding between orbital 10 from Au^+^ and orbital 39 from [SC(NH_2_)_2_]_2_. For Au(SCN)_2_^−^, σ-bonding between orbital 10 from Au^+^ and orbital 27 from (SCN)_2_^2−^ forms the orbital 37 (HOMO-2, σ_g_), which contributes the most for the electrons donated from (SCN)_2_^2−^ to Au^+^. For AuCl_2_^−^, σ-bonds in orbital 25 (HOMO-2, σ_g_) and orbital 20 (HOMO-7, σ_u_) mainly contribute to the σ-donation between Au^+^ and Cl_2_^2–^. Orbital 10 from Au^+^ and orbital 13 from Cl_2_^2−^ compose orbital 25, and orbital 12 from Au^+^ and orbital 18 from Cl_2_^2–^ constitute orbital 20.

**Figure 4:**
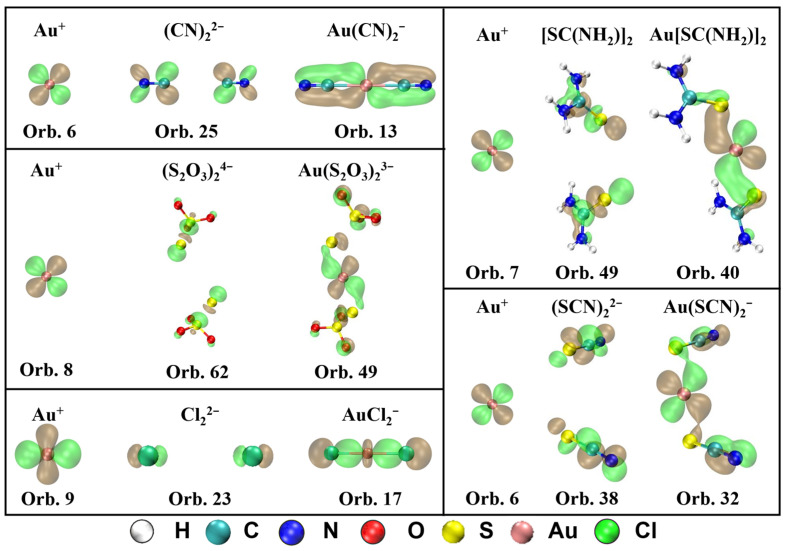
MOs that contribute to π-backdonation and their orbital composition from Au^+^ and ligands for Au(CN)_2_^−^, Au(S_2_O_3_)_2_^3−^, Au[SC(NH_2_)_2_]_2_^+^, Au(SCN)_2_**^−^**, and AuCl_2_**^−^** (isosurface = 0.04 a.u.).

The MOs that contribute to π-backdonation and their orbital composition from Au^+^ and ligands are displayed in Figure 4. It shows that d orbitals from Au^+^ participate in the formation of the π bond in all five complexes. For Au(CN)_2_^−^, the π-backdonation is mainly generated in orbital 13, which is composed of orbital 6 (5d) in Au^+^ and orbital 25 in (CN)_2_^2−^. For Au(S_2_O_3_)_2_^3−^, orbital 49 is in charge of the π-backdonation from Au^+^ to ligands, which is mainly composed of orbital 8 (5d) in Au^+^ and orbital 62 in (S_2_O_3_)_2_^4−^. For Au[SC(NH_2_)_2_]_2_^+^, the π-backdonation from Au^+^ to ligands occurs in orbital 40, which is the combination of orbital 7 (5d) in Au^+^ and orbital 49 in [SC(NH_2_)_2_]_2_. For Au(SCN)_2_^−^, the π-backdonation takes place from orbital 6 in Au^+^ to orbital 38 in (SCN)_2_^2−^, which forms orbital 32 in the complex. For AuCl_2_^−^, orbital 9 (5d) in Au^+^ and orbital 23 in Cl_2_^2−^ forms bonding orbital 17 in the complex, which is in charge with the π-backdonation from metal to ligands.

In the complexes, σ-bonds in those orbitals are all linear or nearly linear. The Au 5d orbitals transform as σ_g_ + π_g_ + δ_g_ in D_∞h_ symmetry. Owing to the strong relativistic effects in gold, the ${\mathrm{sd}}_{z^{2}}$ hybridization is enhanced [38]. The Au 5d atomic orbitals would not be involved in net bonding with main-group elements if there were no sd hybridizations. The sd hybridization results in two hybrid orbitals, which are 180° relative to each other. The geometrical characters of the two sd hybrid orbitals lead to the most effective overlaps with the AO in ligands. The linear geometry results in the maximum overlap between the AOs and the reduction in repulsion between the two negatively charged ligands [27], which is beneficial to stabilize the complexes.

The CDA results give an important insight into the orbital interaction and electron transfer between Au^+^ and ligands. Based on the donation and backdonation analysis in Au-C (Au(CN)_2_^−^), Au-S (Au(S_2_O_3_)_2_^3−^, Au[SC(NH_2_)_2_]_2_^+^, Au(SCN)_2_^−^), and Au-Cl (AuCl_2_^−^), the strength of σ-bonds for the complexes can be explained. From the detailed analysis of orbital composition, the composition and shapes of the σ-bonds and π-backdonation bonds are demonstrated. 

### 2.3. Chemical Bonding Analyses

The natural bond orbital (NBO) and natural resonance theory (NRT) analyses were performed to illustrate the participation of atomic orbitals (AOs) in the bonding orbitals of the complexes, which are listed in Table 5. The NBO results demonstrate significant s-d hybridization in Au^+^ for all five complexes. Additionally, except for Au(CN)_2_^−^, a small amount of orbital p (0.01) also participates in the hybridization. For complexes Au(CN)_2_^−^, Au(S_2_O_3_)_2_^3−^, Au[SC(NH_2_)_2_]_2_^+^, Au(SCN)_2_**^−^**, and AuCl_2_**^−^**, the percentage of Au^+^ is similar, which is around 24~25%, while that in AuCl_2_**^−^** is less, which is no more than 20%. 

Natural resonance theory (NRT) is a theoretical method based on quantum chemical calculations to describe molecules with significant resonance structures in terms of classic valence-bond concepts [39]. The NRT approach allows the expansion of total wave function in terms of chemically intuitive resonance structures [40].

The NRT analysis indicates the covalent and ionic contribution to the Au-L bond. For complexes Au(CN)_2_^−^, Au(S_2_O_3_)_2_^3−^, Au[SC(NH_2_)_2_]_2_^+^, and Au(SCN)_2_**^−^**, they have similar covalent contribution, which is above 0.4, showing covalent character. While for AuCl_2_**^−^**, its covalent bond order is 0.176, which shows more ionic character due to the large electronegativity of Cl_2_^2−^. More covalent contribution indicates that the interaction between metal and ligands is stronger, namely, the bonds would be more stable. For the three complexes containing the Au-S bond, their covalent contribution slightly decreases from Au(S_2_O_3_)_2_^3−^, Au[SC(NH_2_)_2_]_2_^+^, to Au(SCN)_2_**^−^**, which corresponds to their stability.

Electron localization function (ELF) measures the excess of kinetic energy density due to the Pauli repulsion. In the region of space where the Pauli repulsion is strong, ELF is close to 1, whereas where the probability of finding the same-spin electrons close together is high, ELF tends to 0. Regions where the value of ELF is close to 1 correspond to well-localized electrons [41]. ELF is a chemically intuitive tool to estimate the probability of finding electron pairs in space, and an ELF analysis can show the difference among covalent bonds, ionic bonds, and lone pairs [39].

**Figure 5:**
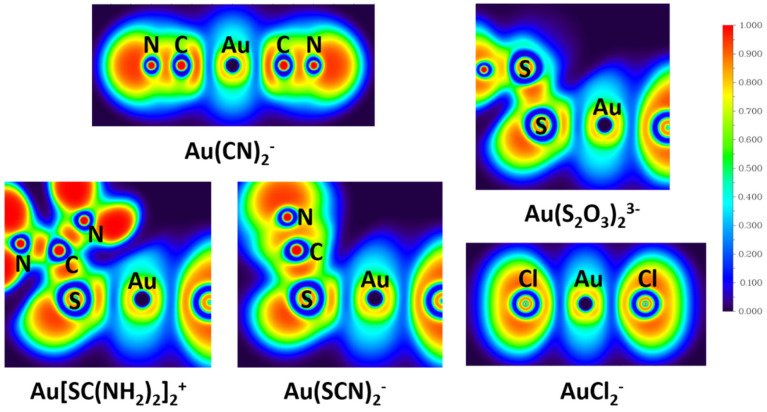
The electron localization functions (ELFs) of Au(CN)_2_^−^, Au(S_2_O_3_)_2_^3−^, Au[SC(NH_2_)_2_]_2_^+^, Au(SCN)_2_**^−^**, and AuCl_2_**^−^** derived from TPSSh/aug-cc-pVTZ calculation.

The calculated ELFs shown in Figure 5 reveal the distributions of the electron pairs in the real space of gold complexes. It is revealed from the ELF analyses that electron pairing densities in the Au-C bond in Au(CN)_2_^−^ and Au–S bonds in Au(S_2_O_3_)_2_^3−^, Au[SC(NH_2_)_2_]_2_^+^, and Au(SCN)_2_^−^ are all around 0.3, while that in the Au-Cl bond in AuCl_2_^−^ is ~0.2. Since a high value of ELF corresponds to well-localized electrons, the electrons in Au-C bonds (Au(CN)_2_^−^) and Au-S bonds (Au(S_2_O_3_)_2_^3−^, Au[SC(NH_2_)_2_]_2_^+^, and Au(SCN)_2_^−^) are more localized than that in Au-Cl bonds (AuCl_2_^−^). Localized electrons are associated with covalent bonds, so Au-C bonds in Au(CN)_2_^−^ and Au-S bonds in Au(S_2_O_3_)_2_^3−^, Au[SC(NH_2_)_2_]_2_^+^, and Au(SCN)_2_^−^ show more covalent character than Au-Cl bonds in AuCl_2_^−^, which is in accord with the NRT analyses results.

NBO analysis supports the information of orbital composition. The NRT and ELF results demonstrate the bond properties between metal and ligands, which gives an important insight into the special stability of Au-CN and Au-S bonds. Compared with Cl^−^, ligands CN^−^, S_2_O_3_^2−^, SC(NH_2_)_2_, and SCN^−^ have a very large covalent contribution to the coordinate bond with Au^+^, which explains the small stability constant of AuCl_2_^−^.

### 2.4. Energy Decomposition Analysis (EDA)

Bonding energy is an important criterion for the stability of a chemical bond. The decomposition of the bonding energy between Au and the ligand provides further insight into the nature of the Au–ligand bonding. According to energy decomposition analysis (EDA), the interaction energy between two fragments can be decomposed into the sum of electrostatic interactions, Pauli repulsion, and the orbital interaction energy [26]:

(6) $${\Delta E}_{\mathrm{bonding}}={\Delta E}_{\mathrm{electrostatic}}+{\Delta E}_{\mathrm{pauli}}+{\Delta E}_{\mathrm{orbint}}={\Delta E}_{\mathrm{steric}}+{\Delta E}_{\mathrm{orbint}}$$

The steric interaction energy is the sum of the electrostatic interaction energy and Pauli repulsion. The orbital interaction can be viewed as an electron transfer from one fragment to the other or as the orbital mixing between two fragments, which indicates the degree of covalency between the fragments. An ionic bond can be described as the interaction between two frozen densities plus intramolecular relaxations, which is ΔE_steric_ in EDA. Therefore, the orbital interaction energy in the total bonding energy reflects the degree of covalency in the chemical bond between two atoms or fragments.

The EDA results for one-ligand deposition are shown in Table 6. It displays that the steric interaction is positive, indicating that the interaction introduced by steric is repulsive. The value of steric repulsion is AuCl_2_^−^ < Au(CN)_2_^−^ < Au(SCN)_2_**^−^** < Au[SC(NH_2_)_2_]_2_^+^ < Au(S_2_O_3_)_2_^3−^, which can be explained by the ligand size and the symmetry of the complex. The ligand size increases from Cl^−^, CN^−^, SCN^−^, SC(NH_2_)_2_, to S_2_O_3_^4−^. Additionally, for AuCl_2_^−^ and Au(CN)_2_^−^, all atoms are in the same surface and the complex is in a linear shape. However, for Au(SCN)_2_**^−^**, Au[SC(NH_2_)_2_]_2_^+^,and Au(S_2_O_3_)_2_^3−^, the metal atom and ligand atoms are not in the same surface and the S-Au-S bond is not linear, which also lead to the steric repulsion; the orbital interactions are all negative, which indicates the attractive interaction between Au^+^ and ligands. The value of orbital interaction increases from Au(CN)_2_^−^, Au(S_2_O_3_)_2_^3−^, Au[SC(NH_2_)_2_]_2_^+^, Au(SCN)_2_**^−^**, to AuCl_2_**^−^** meaning the degree of covalency between the fragments decreases. Combined the steric interaction and orbital interaction, the total bonding energy is negative, revealing the attraction between L-Au and ligand. In addition, it also indicates that the degree of covalency is larger than that of ionic. The value of total interaction is in the order Au(CN)_2_^−^ > Au(S_2_O_3_)_2_^3−^ > Au[SC(NH_2_)_2_]_2_^+^ > Au(SCN)_2_^−^, except AuCl_2_**^−^** whose total interaction is a little larger than that of Au(SCN)_2_^−^ because of its low steric interaction.

The EDA results for two-ligand deposition are illustrated in Table 7, which has similar tendency with that in Table 6. The steric interaction is positive, while the orbital interactions are all negative. Compared with the values in Table 6, it is found that the energy for two-ligands deposition is more than twice of that for one-ligand deposition. The value of orbital interaction also increases from Au(CN)_2_^−^, Au(S_2_O_3_)_2_^3−^, Au[SC(NH_2_)_2_]_2_^+^, Au(SCN)_2_^−^, to AuCl_2_^−^, meaning the degree of covalency between the fragments decreases. The value of total interaction is also in the order Au(CN)_2_^−^ > Au(S_2_O_3_)_2_^3−^ > Au[SC(NH_2_)_2_]_2_^+^ > Au(SCN)_2_^−^ > AuCl_2_^−^.

The covalency in the coordinate bond and the total interaction value are related to the stability of the complex. Based on the results in Table 6 and Table 7, it can be concluded that the EDA results are consistent with the stability of the five complexes, which is Au(CN)_2_^−^ > Au(S_2_O_3_)_2_^3−^ > Au[SC(NH_2_)_2_]_2_^+^ > Au(SCN)_2_^−^ > AuCl_2_^−^.

## 3. Computational Methods

All DFT calculations were performed in the Gaussian 16 [42] program by using hybrid meta-GGA functional TPSSh [43] with aug-cc-pVTZ basis set [44,45,46] for nonmetallic atoms and Stuttgart pseudopotentials ECP60MDF with corresponding augmented valence basis aug-cc-pVTZ-PP for metallic atom Au [47,48]. For comparison, all complexes were also optimized at high-quality B2PLYP/def2-QZVP level [49,50]. The polarizable continuum model (PCM) model [51] was used to incorporate solvation effects with water for all calculations. All of the geometries were confirmed to be minima by performing a vibrational frequency calculation and had no imaginary frequency.

The natural bond orbital (NBO) and natural resonance theory (NRT) calculations were implemented in the NBO 5.0 package [52]. The charge decomposition analysis (CDA) [33,53], electron localization function (ELF) [54], and energy decomposition analysis (EDA) calculations were performed in Multiwfn 3.8 (dev) software [55].

## 4. Conclusions

The charge decomposition analysis (CDA), natural bond orbital (NBO), natural resonance theory (NRT), electron localization function (ELF), and energy decomposition analysis (EDA) for complexes Au(CN)_2_^−^, Au(S_2_O_3_)_2_^3−^, Au[SC(NH_2_)_2_]_2_^+^, Au(SCN)_2_^−^, and AuCl_2_^−^ were performed by DFT calculation, based on which their structure information, electron transferring properties, orbital interaction, chemical bond composition, and bond energy were comprehensively elaborated. This study aimed to support the bonding information to the potential gold leaching reagent. The following conclusions can be drawn:

(1) Based on the CDA results, the orbital interaction and electron transferring between Au^+^ and ligands for Au(CN)_2_^−^, Au(S_2_O_3_)_2_^3−^, Au[SC(NH_2_)_2_]_2_^+^, Au(SCN)_2_^−^, and AuCl_2_^−^ are interpreted. There is not only σ-donation from ligand to Au^+^, but also electron backdonation from Au^+^ to ligands, which strengthens the coordinate bond between them. The percentage of π-backdonation in Au(CN)_2_^−^ is the largest contributing to its high stability.
(2) From the perspective of NRT and ELF, compared with Cl^−^, ligands CN^−^, S_2_O_3_^2−^, SC(NH_2_)_2_, and SCN^−^ have very large covalent contribution to the coordinate bond with Au+, which explains the special stability of Au-CN and Au-S bonds.
(3) The decomposition of the bonding energy between Au and the ligand provides further insight into the nature of the Au–ligand bonding. The EDA results show the increased value of orbital interaction from Au(CN)_2_^−^, Au(S_2_O_3_)_2_^3−^, Au[SC(NH_2_)_2_]_2_^+^, Au(SCN)_2_^−^, to AuCl_2_^−^, corresponding to the decreased degree of covalency in Au–ligand bonding, which interprets the stability of the five complexes: Au(CN)_2_^−^ > Au(S_2_O_3_)_2_^3−^ > Au[SC(NH_2_)_2_]_2_^+^ > Au(SCN)_2_^−^ > AuCl_2_^−^.

## Figures and Tables

**Table 1:** Stability constants and standard reduction potentials for Au(CN)_2_^−^, Au(S_2_O_3_)_2_^3−^, Au[SC(NH_2_)_2_]_2_^+^, Au(SCN)_2_^−^, and AuCl_2_^−^ [1,5,14].

Complex	Au(CN)_2_^−^	Au(S_2_O_3_)_2_^3−^	Au[SC(NH_2_)_2_]_2_^+^	Au(SCN)_2_^−^	AuCl_2_^−^
logβ_i_	38.3	28.7	21.3	17.1	9.1
E0/V	−0.57	0.17	0.35	0.66	1.11

**Table 2:** The geometry and structural parameters of Au(CN)_2_^−^, Au(S_2_O_3_)_2_^3−^, Au[SC(NH_2_)_2_]_2_^+^, Au(SCN)_2_^−^, and AuCl_2_^−^ at TPSSh/aug-cc-pVTZ (a) and B2PLYP/def2-QZVP (b) levels. The lengths are given in Å and angles are given in degree.

Complex	Symm.	R(Au − X)(X = C, Cl, S)		∠(X – Au − X)(X = C, Cl, S)	
		a	b	a	b
Au(CN)_2_^−^	D_∞h_	1.987	1.987	180.00	180.00
Au(S_2_O_3_)_2_^3−^	C_2_	2.299	2.297	176.45	176.86
Au[SC(NH_2_)_2_]_2_^+^	C_2_	2.304	2.299	176.18	176.15
Au(SCN)_2_^−^	C_2_	2.310	2.305	176.94	177.16
AuCl_2_^−^	D_∞h_	2.291	2.287	180.00	180.00

**Table 3:** The atomic charges on Au and ligand atoms for Au(CN)_2_^−^, Au(S_2_O_3_)_2_^3−^, Au[SC(NH_2_)_2_]_2_^+^, Au(SCN)_2_^−^, and AuCl_2_^−^.

Complex	Atom	Charge			
		Hirshfeld [28]	ADCH [29]	CM5 [30]	CHELPG [31]
Au(CN)_2_^−^	Au	0.089	0.100	0.207	0.197
	C	−0.173	−0.077	−0.109	−0.020
Au(S_2_O_3_)_2_^3−^	Au	−0.092	0.031	0.042	0.290
	S	−0.409	−0.502	−0.469	−0.727
Au[SC(NH_2_)_2_]_2_^+^	Au	0.135	0.153	0.271	0.427
	S	−0.178	−0.224	−0.247	−0.438
Au(SCN)_2_^−^	Au	0.066	0.267	0.195	0.262
	S	−0.178	−0.238	−0.252	−0.393
AuCl_2_^−^	Au	−0.036	−0.004	0.056	0.270
	Cl	−0.482	−0.498	−0.528	−0.636

**Table 4:** Relative contributions to the coordinate bond and percentage contribution of the backdonation relative to the donation from CDA of Hartree–Fock wavefunctions at TPSSh/aug-cc-pVTZ level.

Complex	Donation	Backdonation	Backdonation/Donation (%)
Au(CN)_2_^−^	0.8035	0.1464	18.22
Au(S_2_O_3_)_2_^3−^	0.5294	0.0829	15.66
Au[SC(NH_2_)_2_]_2_^+^	0.4458	0.0722	16.20
Au(SCN)_2_^−^	0.6673	0.0938	14.06
AuCl_2_^−^	0.6226	0.0453	7.28

**Table 5:** Natural bond orbital (NBO) analysis and natural resonance theory (NRT) bond order (BO) of Au(CN)_2_^−^, Au(S_2_O_3_)_2_^3−^, Au[SC(NH_2_)_2_]_2_^+^, Au(SCN)_2_**^−^**, and AuCl_2_**^−^**.

Complex	NBO Analysis	NRT BO		
		Covalent	Ionic	Covalent/Total
Au(CN)_2_^−^	25.37% (sd^0.27^)_Au_ + 74.63% (sp^0.87^)_C_	0.215	0.312	0.408
Au(S_2_O_3_)_2_^3−^	24.01% (sp^0.01^d^0.18^)_Au_ + 75.99% (sp^5.82^d^0.04^)_S_	0.215	0.257	0.456
Au[SC(NH_2_)_2_]_2_^+^	24.70% (sp^0.01^d^0.18^)_Au_ + 75.30% (sp^6.54^d^0.04^)_S_	0.216	0.265	0.449
Au(SCN)_2_^−^	24.22% (sp^0.01^d^0.18^)_Au_ + 75.78% (sp^9.76^d^0.05^)_S_	0.194	0.253	0.434
AuCl_2_^−^	19.41% (sp^0.01^d^0.21^)_Au_ + 80.59% (sp^6.61^d^0.02^)_Cl_	0.176	0.824	0.176

**Table 6:** Energy decomposition analyses for [XAuX]^−^ → AuX + X^−^ with the water model. The results are based on the TPSSh/aug-cc-pVTZ calculations. All energies are given in eV.

Complex	Steric Interaction	Orbital Interaction	Total	Orb./Total
Au(CN)_2_^−^	1.32	−4.09	−2.77	1.477
Au(S_2_O_3_)_2_^3−^	1.49	−3.64	−2.14	1.696
Au[SC(NH_2_)_2_]_2_^+^	1.46	−3.43	−1.97	1.740
Au(SCN)_2_^−^	1.34	−3.17	−1.83	1.737
AuCl_2_^−^	0.83	−2.70	−1.86	1.446

**Table 7:** Energy decomposition analyses for [XAuX]^−^ → X^−^ + Au+ X^−^ with the water model. The results are based on the TPSSh/aug-cc-pVTZ calculations. All energies are given in eV.

Complex	Steric Interaction	Orbital Interaction	Total	Orb./Total
Au(CN)_2_^−^	3.41	−9.37	−5.97	1.571
Au(S_2_O_3_)_2_^3−^	2.68	−7.84	−5.16	1.520
Au[SC(NH_2_)_2_]_2_^+^	3.17	−7.43	−4.26	1.744
Au(SCN)_2_^−^	2.84	−6.90	−4.05	1.701
AuCl_2_^−^	2.35	−6.22	−3.87	1.606

## Data Availability

Not applicable.

## References

[1] Li J., Miller J.D. (2006). A Review of Gold Leaching in Acid Thiourea Solutions. Miner. Process. Extr. Metall. Rev..

[2] Li L., Pan C., Shan J., She W., You X., Ji C., Gao Q. (2020). Pit-Induced Electrochemical Layer Dissolution and Wave Propagation on an Au(111) Surface in an Acidic Thiourea Solution. J. Phys. Chem. C.

[3] Zhang Y., Xu B., Cui M., Li Q., Liu X., Jiang T., Lyu X. (2021). Thiosulfate leaching of gold catalyzed by hexaamminecobalt(III): Electrochemical behavior and mechanisms. Electrochim. Acta.

[4] Li W.-J., Zhou H., Bai A.-P., Song Y.-S., Cai L.-L., Zheng S.-L., Zhang Q.-D., Cao S. (2020). Electrochemical adsorption and passivation on gold surface in alkaline thiourea solutions. Rare Met..

[5] Birich A., Stopic S., Friedrich B. (2019). Kinetic Investigation and Dissolution Behavior of Cyanide Alternative Gold Leaching Reagents. Sci. Rep..

[6] Zhang X.M., Senanayake G. (2016). A Review of Ammoniacal Thiosulfate Leaching of Gold: An Update Useful for Further Research in Non-cyanide Gold Lixiviants. Miner. Process. Extr. Metall. Rev..

[7] Whitehead J., Zhang J., McCluskey A., Lawrance G. (2009). Comparative leaching of a sulfidic gold ore in ionic liquid and aqueous acid with thiourea and halides using Fe(III) or HSO5−oxidant. Hydrometallurgy.

[8] Azizitorghabeh A., Wang J., Ramsay J.A., Ghahreman A. (2021). A review of thiocyanate gold leaching—Chemistry, thermodynamics, kinetics and processing. Miner. Eng..

[9] Ilyas S., Srivastava R.R., Kim H. (2021). Gold recovery from secondary waste of PCBs by electro-Cl_2_ leaching in brine solution and solvo-chemical separation with tri-butyl phosphate. J. Clean. Prod..

[10] Senanayake G. (2005). Gold leaching by thiosulphate solutions: A critical review on copper(II)–thiosulphate–oxygen interactions. Miner. Eng..

[11] Grosse A.C., Dicinoski G.W., Shaw M.J., Haddad P. (2003). Leaching and recovery of gold using ammoniacal thiosulfate leach liquors (a review). Hydrometallurgy.

[12] Sitando O., Senanayake G., Dai X., Nikoloski A., Breuer P. (2018). A review of factors affecting gold leaching in non-ammoniacal thiosulfate solutions including degradation and in-situ generation of thiosulfate. Hydrometallurgy.

[13] Xu B., Kong W., Li Q., Yang Y., Jiang T., Liu X. (2017). A Review of Thiosulfate Leaching of Gold: Focus on Thiosulfate Consumption and Gold Recovery from Pregnant Solution. Metals.

[14] Aylmore M.G., Muir D.M. (2001). Thiosulfate leaching of gold—A review. Miner. Eng..

[15] Yu B., Liu Y., Peng X., Hua S., Zhou G., Yan K., Liu Y. (2020). Synthesis, characterization, and antitumor properties of Au(i)-thiourea complexes. Metallomics.

[16] Yang X., Moats M.S., Miller J.D., Wang X., Shi X., Xu H. (2011). Thiourea–thiocyanate leaching system for gold. Hydrometallurgy.

[17] Kholmogorov A., Kononova O., Pashkov G., Kononov Y. (2002). Thiocyanate solutions in gold technology. Hydrometallurgy.

[18] Azizitorghabeh A., Mahandra H., Ramsay J., Ghahreman A. (2021). Gold leaching from an oxide ore using thiocyanate as a lixiviant: Process optimization and kinetics. ACS Omega.

[19] Nikoloski A., Stockton B. Application of alternative lixiviants for secondary heap leaching of gold. Proceedings of the 7th Mill Operators Conference.

[20] Dönmez B., Sevim F., Çolak S. (2001). A Study on Recovery of Gold from Decopperized Anode Slime. Chem. Eng. Technol..

[21] Wang S., Li L., Wang H., Wu G.-D. (2020). Extraction of platinum and gold from copper anode slimes by a process of chlorinating roasting followed by chlorinating leaching. J. Min. Met. Sect. B Met..

[22] Rodríguez M., Ayala L., Robles P., Sepúlveda R., Torres D., Carrillo-Pedroza F.R., Jeldres R.I., Toro N. (2020). Leaching Chalcopyrite with an Imidazolium-Based Ionic Liquid and Bromide. Metals.

[23] Li G.-Z., Kou J., Xing Y., Hu Y., Han W., Liu Z.-Y., Sun C.-B. (2021). Gold-leaching performance and mechanism of sodium dicyanamide. Int. J. Miner. Met. Mater..

[24] Liu Z., Kou J., Xing Y., Sun C. (2021). Recovery of Gold from Ore with Potassium Ferrocyanide Solution under UV Light. Minerals.

[25] Pyykkö P., Atsumi M. (2009). Molecular single-bond covalent radii for elements 1-118. Chemistry.

[26] Xiong X.G., Wang Y.L., Xu C.Q., Qiu Y.H., Wang L.S., Li J. (2015). On the gold-ligand covalency in linear [AuX_2_]^(−)^ complexes. Dalton Trans..

[27] Xu C.Q., Xiong X.G., Li W.L., Li J. (2016). Periodicity and Covalency of [MX_2_]^−^ (M=Cu, Ag, Au, Rg;X=H, Cl, CN) Complexes. Eur. J. Inorg. Chem..

[28] Hirshfeld F.L. (1977). Bonded-atom fragments for describing molecular charge densities. Theor. Chim. Acta.

[29] Lu T., Chen F. (2012). Atomic dipole moment corrected Hirshfeld population method. J. Theor. Comput. Chem..

[30] Marenich A.V., Jerome S.V., Cramer C.J., Truhlar D.G. (2012). Charge Model 5: An Extension of Hirshfeld Population Analysis for the Accurate Description of Molecular Interactions in Gaseous and Condensed Phases. J. Chem. Theory Comput..

[31] Breneman C.M., Wiberg K. (1990). Determining atom-centered monopoles from molecular electrostatic potentials. The need for high sampling density in formamide conformational analysis. J. Comput. Chem..

[32] Chatt J., Duncanson L.A. (1953). 586. Olefin co-ordination compounds. Part III. Infrared spectra and structure: Attempted preparation of acetylene complexes. J. Chem. Soc..

[33] Dapprich S., Frenking G. (1995). Investigation of donor-acceptor interactions: A charge decomposition analysis using fragment molecular orbitals. J. Phys. Chem..

[34] Hernández M.G., Beste A., Frenking G., Illas F. (2000). Charge decomposition analysis of the chemisorption bond. Chem. Phys. Lett..

[35] Ehlers A.W., Dapprich S., Vyboishchikov S.F., Frenking G. (1996). Structure and Bonding of the Transition-Metal Carbonyl Complexes M (CO) 5L (M= Cr, Mo, W) and M (CO) 3L (M= Ni, Pd, Pt; L= CO, SiO, CS, N_2_, NO^+^, CN^−^, NC^−^, HCCH, CCH_2_, CH_2_, CF_2_, H_2_). Organometallics.

[36] Szilagyi R.K., Frenking G.J.O. (1997). Structure and Bonding of the Isoelectronic Hexacarbonyls [Hf(CO)_6_]^2−^, [Ta(CO)_6_]^−^, W(CO)_6_, [Re(CO)_6_]^+^, [Os(CO)_6_]^2+^, and [Ir(CO)_6_]^3+^: A Theoretical Study. Organometallics.

[37] Crabtree R.H. (2009). The Organometallic Chemistry of the Transition Metals.

[38] Pyykko P.J.C.R. (1988). Relativistic effects in structural chemistry. Chem. Rev..

[39] Ning C.G., Xiong X.G., Wang Y.L., Li J., Wang L.S. (2012). Probing the electronic structure and chemical bonding of the “staple” motifs of thiolate gold nanoparticles: Au(SCH_3_)^2−^ and Au_2_(SCH_3_)_3_. Phys. Chem. Chem. Phys..

[40] Glendening E.D., Weinhold F. (1998). Natural resonance theory: I. General formalism. J. Comput. Chem..

[41] Poater J., Duran M., Solà M., Silvi B. (2005). Theoretical Evaluation of Electron Delocalization in Aromatic Molecules by Means of Atoms in Molecules (AIM) and Electron Localization Function (ELF) Topological Approaches. Chem. Rev..

[42] Frisch M.E., Trucks G.W., Schlegel H.B., Scuseria G.E., Robb M.A., Cheeseman J.R., Scalmani G., Barone V.P.G.A., Petersson G.A., Nakatsuji H.J.R.A. (2016). Gaussian 16.

[43] Tao J., Perdew J.P., Staroverov V.N., Scuseria G.E. (2003). Climbing the Density Functional Ladder: Nonempirical Meta–Generalized Gradient Approximation Designed for Molecules and Solids. Phys. Rev. Lett..

[44] Dunning T.H. (1989). Gaussian basis sets for use in correlated molecular calculations. I. The atoms boron through neon and hydrogen. J. Chem. Phys..

[45] Woon D.E., Dunning T.H. (1993). Gaussian basis sets for use in correlated molecular calculations. III. The atoms aluminum through argon. J. Chem. Phys..

[46] Wilson A.K., Woon D.E., Peterson K.A., Dunning T.H. (1999). Gaussian basis sets for use in correlated molecular calculations. IX. The atoms gallium through krypton. J. Chem. Phys..

[47] Figgen D., Rauhut G., Dolg M., Stoll H. (2005). Energy-consistent pseudopotentials for group 11 and 12 atoms: Adjustment to multi-configuration Dirac–Hartree–Fock data. Chem. Phys..

[48] Peterson K.A., Puzzarini C. (2005). Systematically convergent basis sets for transition metals. II. Pseudopotential-based correlation consistent basis sets for the group 11 (Cu, Ag, Au) and 12 (Zn, Cd, Hg) elements. Theor. Chem. Acc..

[49] Grimme S. (2006). Semiempirical hybrid density functional with perturbative second-order correlation. J. Chem. Phys..

[50] Weigend F., Ahlrichs R. (2005). Balanced basis sets of split valence, triple zeta valence and quadruple zeta valence quality for H to Rn: Design and assessment of accuracy. Phys. Chem. Chem. Phys..

[51] Cammi R., Tomasi J. (1995). Remarks on the use of the apparent surface charges (ASC) methods in solvation problems: Iterative versus matrix-inversion procedures and the renormalization of the apparent charges. J. Comput. Chem..

[52] Weinhold F., Glendening E.D. (2001). NBO 5.0 Program Manual: Natural Bond Orbital Analysis Programs.

[53] Xiao M., Lu T. (2015). Generalized charge decomposition analysis (GCDA) method. J. Adv. Phys. Chem..

[54] Becke A.D., Edgecombe K.E. (1990). A simple measure of electron localization in atomic and molecular systems. J. Chem. Phys..

[55] Lu T., Chen F. (2012). Multiwfn: A multifunctional wavefunction analyzer. J. Comput. Chem..

